# Microcystin-LR Detected in a Low Molecular Weight Fraction from a Crude Extract of *Zoanthus sociatus*

**DOI:** 10.3390/toxins9030089

**Published:** 2017-03-01

**Authors:** Dany Domínguez-Pérez, Armando Alexei Rodríguez, Hugo Osorio, Joana Azevedo, Olga Castañeda, Vítor Vasconcelos, Agostinho Antunes

**Affiliations:** 1 CIIMAR/CIMAR, Interdisciplinary Centre of Marine and Environmental Research, University of Porto, Terminal de Cruzeiros do Porto de Leixões, Av. General Norton de Matos, s/n, 4450-208 Porto, Portugal; danydguezperez@gmail.com (D.D.-P.); joana_passo@hotmail.com (J.A.); vmvascon@fc.up.pt (V.V.); 2 Department of Biology, Faculty of Sciences, University of Porto, Rua do Campo Alegre, s/n, 4169-007 Porto, Portugal; 3 Department of Experimental and Clinical Peptide Chemistry, Hanover Medical School (MHH), Feodor-Lynen-Straße 31, D-30625 Hannover, Germany; aara259@gmail.com; 4 i3S - Instituto de Investigação e Inovação em Saúde, Universidade do Porto, Rua Alfredo Allen, 208, 4200-135 Porto, Portugal; hosorio@ipatimup.pt; 5 Ipatimup, Institute of Molecular Pathology and Immunology of the University of Porto, Rua Júlio Amaral de Carvalho, 45, 4200-135 Porto, Portugal; 6 Department of Pathology and Oncology, Faculty of Medicine, University of Porto, Al. Prof. Hernâni Monteiro, 4200-319 Porto, Portugal; 7 Faculty of Biology, University of La Habana, 25 St 455, CP 10400 La Habana, Cuba; castañedapasaron@gmail.com

**Keywords:** microcystins, MC-LR, *Zoanthus sociatus*, zoanthidea, cnidarian, Sephadex G50, MALDI-TOF/TOF

## Abstract

Cnidarian constitutes a great source of bioactive compounds. However, research involving peptides from organisms belonging to the order Zoanthidea has received very little attention, contrasting to the numerous studies of the order Actiniaria, from which hundreds of toxic peptides and proteins have been reported. In this work, we performed a mass spectrometry analysis of a low molecular weight (LMW) fraction previously reported as lethal to mice. The low molecular weight (LMW) fraction was obtained by gel filtration of a *Zoanthus sociatus* (order Zoanthidea) crude extract with a Sephadex G-50, and then analyzed by matrix-assisted laser desorption/ionization time-of-flight/time-of-flight (MALDI-TOF/TOF) mass spectrometry (MS) in positive ion reflector mode from *m*/*z* 700 to *m*/*z* 4000. Afterwards, some of the most intense and representative MS ions were fragmented by MS/MS with no significant results obtained by Protein Pilot protein identification software and the Mascot algorithm search. However, microcystin masses were detected by mass-matching against libraries of non-ribosomal peptide database (NORINE). Subsequent reversed-phase C18 HPLC (in isocratic elution mode) and mass spectrometry analyses corroborated the presence of the cyanotoxin Microcystin-LR (MC-LR). To the best of our knowledge, this finding constitutes the first report of MC-LR in *Z. sociatus*, and one of the few evidences of such cyanotoxin in cnidarians.

## 1. Introduction

Cnidarians represent promising sources of bioactive compounds, which can be of major pharmacological [1] and therapeutic interest [2,3,4]. However, many organisms belonging to the order Actiniaria (sea anemones), of the class Anthozoa, have been so far studied [5,6,7,8]. In contrast, the order Zoanthidea has been scarcely explored for biologically active compounds, although some potent non-peptide toxins, such as palytoxin and its analogues, have been characterized from this order [9,10,11]. Additionally, a peptide exhibiting a reversible delay of tetrodotoxin (TTX)-sensitive sodium channel inactivation, was isolated and characterized from *Palythoa caribaeorum* exudate, but its sequence does not resemble any cnidarian toxin previously reported [12]. In another study the exudate of *P. caribaeourum* provoked reversible delay of the inactivation process of voltage-gated sodium channels (Na_V_1.7), inhibited voltage-gated calcium (Ca_V_2.2) and delayed rectifier (I_DR_) currents of K_V_ channels from rat superior cervical ganglion (SCG) neurons [12]. The matrix-assisted laser desorption/ionization time-of-flight/time-of-flight (MALDI-TOF/TOF) mass spectrometry (MS) analyses provided evidence of low molecular weight peptides involved in such effects on ion channels. It is also noteworthy the transcriptomic analysis on *Protopalythoa variabilis*, at which a transcript encoded a toxin called ShK/Aurelin-like that was toxic to zebrafish embryos [13].

*Zoanthus sociatus* also belongs to the order Zoanthidea. To date, few chemical studies have been reported from this organism, comprising the isolation and characterization of small non-proteinaceous molecules, such as the sterol zoanthosterol [14], as well as the norzoanthamines alkaloids and their analogues [15,16]. In addition to these bioactive compounds, an antifilarial activity from the *Z. sociatus* chloroform/methanol extract was reported [17]. Moreover, only two reports on the toxicity of the crude extract and semi-pure chromatographic fractions have been published. The first study demonstrated that *Z. sociatus* components below 7 kDa are responsible for the inhibition of insulin secretion mediated by Ca^2+^ influx blockade in isolated rat β cells [18]. A further study showed the biological evaluation of a low molecular weight (LMW) Sephadex G-50-chromatographic fraction lethal to mice, presumably by cardiorespiratory arrest [19].

Information provided in previous studies about the molecular mass profile of the low molecular weight (LMW) fraction from *Z. sociatus* showed components in the *m*/*z* range from 700 to 6000 [18,19]. Nonetheless, the mass spectrometry analysis of a LMW fraction obtained by the same methodology showed many *m*/*z* signals below 1000 [19]. Yet, the identification of the main components of the fraction remains to be done, thus, our main goal being the MALDI-TOF/TOF mass spectrometry analysis to characterize the most significant components of a LMW fraction from *Z. sociatus*. First, we performed the acquisition of a MALDI-TOF/TOF MS spectrum of the LMW fraction followed by MS/MS fragmentation of the most significant signals. In general, the obtained data did not allow the identification of sea anemones-like peptide toxins or linear peptides using the Protein Pilot protein identification software or the Mascot algorithm search [20].

On the other hand, *m*/*z* signals from known cyanotoxins were detected by mass-matching against libraries of non-ribosomal peptide database (NORINE) [21,22]. Indeed, within the microcystins masses detected, the *m*/*z* 995.53 matched the expected *m*/*z* signal for microcystin-LR (MC-LR). The presence of MC-LR was further demonstrated by high-resolution mass spectrometry analysis. While microcystins are recognized as freshwater cyanobacterial toxins, the occurrence of microcystins in marine ecosystems is not an isolated fact [23,24,25,26,27]. However, our findings constitute one of the few evidence of such cyanotoxin detected in cnidarians [28], and to the best of our knowledge the first report in *Z. sociatus*. Additionally, this work calls for further attention to the probabilities of water and food contamination by microcystins in tropical regions.

## 2. Results and Discussion

### 2.1. Gel Filtration in Sephadex G-50

The first purification step of the *Z. sociatus* crude extract was achieved by fractionation on Sephadex G-50. As previously described, the gel filtration chromatogram was divided into four fractions [19]. The resulting chromatogram showed the same elution profile of *Z. sociatus* crude extract, obtained by a similar purification protocol on HiLoad 16/20 Superdex 75 column [18]. The third fraction, called Zs G50-III, was then submitted to MALDI-TOF/TOF mass spectrometry analysis, given its lethal effect on mice as previously reported [19].

### 2.2. Zs G50-III MALDI-TOF/TOF Analysis

**Figure 1:**
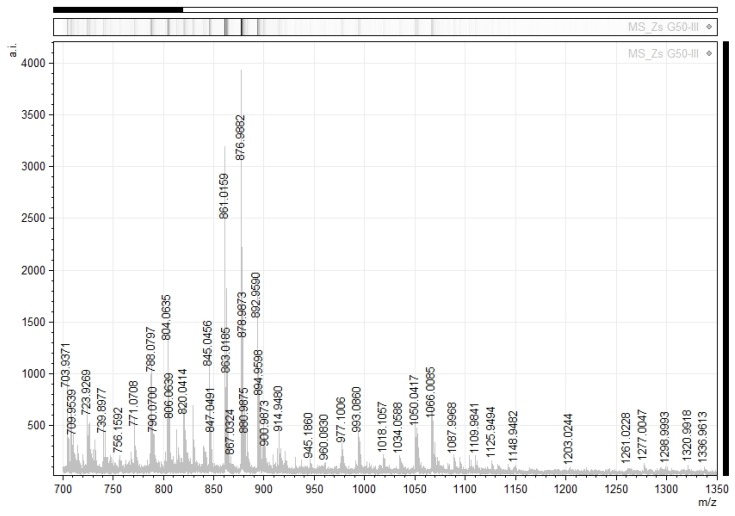
Matrix-assisted laser desorption/ionization time-of-flight/time-of-flight (MALDI-TOF) mass spectrum of the Sephadex G-50 fraction called Zs G50-III. The mass spectrum shows absolute ion intensity (a.i) versus mass-to-charge ratio (*m*/*z*) of Zs G50-III components for the mass range *m*/*z* 700 to 1350.

**Figure 2:**
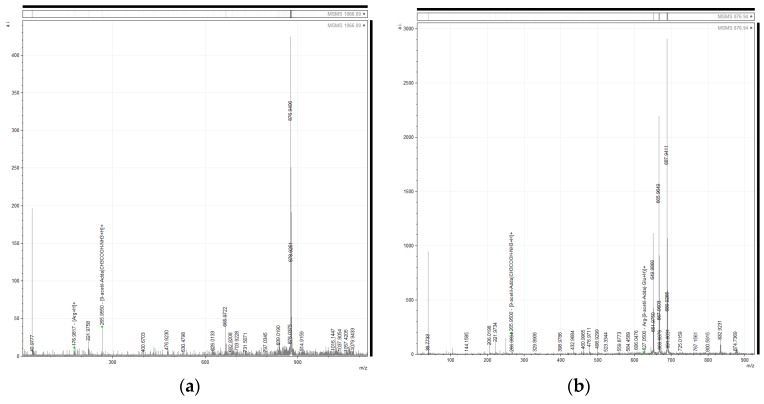
Matrix-assisted laser desorption/ionization time-of-flight/time-of-flight (MALDI-TOF/TOF) mass-spectra of two peaks of interest from fraction Zs G50-III. (**a**) Analysis of *m*/*z* 1066.00 is shown, highlighting the intense signal at *m*/*z* 876.94; and (**b**) analysis of the *m*/*z* 876.94. This signal occurred in the MALDI-TOF/TOF spectrum of *m*/*z* 1066.00, but another signal was also identified at *m*/*z* 265.95.

**Figure 3:**
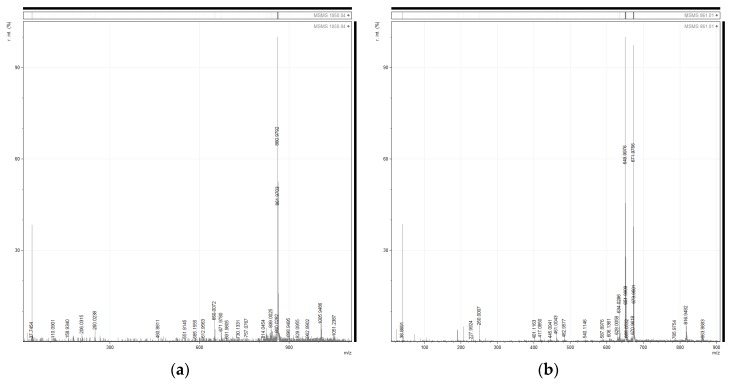
Matrix-assisted laser desorption/ionization time-of-flight/time-of-flight (MALDI-TOF/TOF) analysis of two signals from fraction Zs G50-III mass spectrum. The figure shows the relative intensity (r.int) versus mass-to-charge ratio (*m*/*z*). (**a**) MALDI-TOF/TOF analysis of *m*/*z* 1050.04, where the most intense signal corresponds to *m*/*z* 860.97; and (**b**) MALDI-TOF/TOF analysis of *m*/*z* 861.01.

The selected fraction Zs G50-III was analyzed by MALDI-TOF/TOF MS analysis in positive ion reflector mode in the *m*/*z* range from 700 to 4000. The most intense signal detected in the MALDI-TOF/TOF MS analysis corresponded to *m*/*z* values below 2000, resulting in the detection of 142 signals, ranging from *m*/*z* 703.93 to 1336.96. The highest intensity signals are shown in Figure 1. Afterwards, some of the most intense and representative MS ions corresponding to *m*/*z* 876.98, 861.01, 1050.04, and 1066.00 were fragmented by MS/MS (Figure 2 and Figure 3). Then, all the data generated by the MALDI-TOF/TOF procedure were submitted to search with the Mascot (Matrix-Science, London, UK) algorithm against UniProtKB protein sequence database [29], specifically in the Metazoan and Cnidaria section. Additionally, the spectra were also analyzed with the Protein Pilot protein identification software v4.5 (AB SCIEX). No significant result was obtained by any of the mentioned methods. The fragmentation pattern of the selected ions showed scarce peaks. In fact, the spectra do not resemble those of linear peptides, but seem to be related to cyclo-peptides fragmentation.

On the other hand, cyanotoxins-related masses were detected by peak list mass-matching against libraries of non-ribosomal peptide database (NORINE). Indeed, some of the most intense signals were related to microcystins. The mass *m*/*z* 1066.00 matched a reported microcystin [9-acetyl-Adda5]-MC-RR [30]. Furthermore, the MS/MS analyses of *m*/*z* 1066.00 showed a high intensity signal at *m*/*z* 876.94 (Figure 2). Besides, in the MS/MS spectra of *m*/*z* 1066.00 and *m*/*z* 876.94 signals at *m*/*z* 265.95 and 627.05, were detected, respectively (Figure 2). These masses were previously found in the LC/ESI-Q-ToF-MS/MS spectrum of the microcystin [9-acetyl-Adda5]-MC-RR [30]. Other important diagnostic signals such as *m*/*z* 135 corresponding to 3-amino-9-methoxy-2,6,8-trimethyl-10-phenyl-deca-4,6-dienoic acid (Adda) fragment, or *m*/*z* 163 corresponding to acetyl-Adda side chain were absent or could not be detected because of their low intensity.

In addition, the signal of *m*/*z* 1050.04 matched a reported microcystin, MC-(H_4_) YR [31,32]. Within the most intense signals *m*/*z* 860.97 and *m*/*z* 861.97 were found, resembling isotopic peaks (Figure 3). Similarly, in the MALDI-TOF analysis of fraction Zs G50-III, the *m*/*z* 861.01 was detected as one of the most intense signals. In addition, the fragmentation pattern of *m*/*z* 1050.04 and *m*/*z* 861.01 showed some common signals (Figure 3). Contrasting with the analysis of *m*/*z* 1066.00, it was not possible to match the fragments of *m*/*z* 1050 with known signals from the fragmentation of MC-(H_4_) YR [31]. The diagnostic fragments annotation was hindered because of the limited information from the MC-(H_4_) YR *m*/*z* 1050 spectrum. Moreover, in this case, the spectrum also lacked the microcystins diagnostic signals like Adda fragments *m*/*z* 135 and *m*/*z* 163 [30].

**Figure 4:**
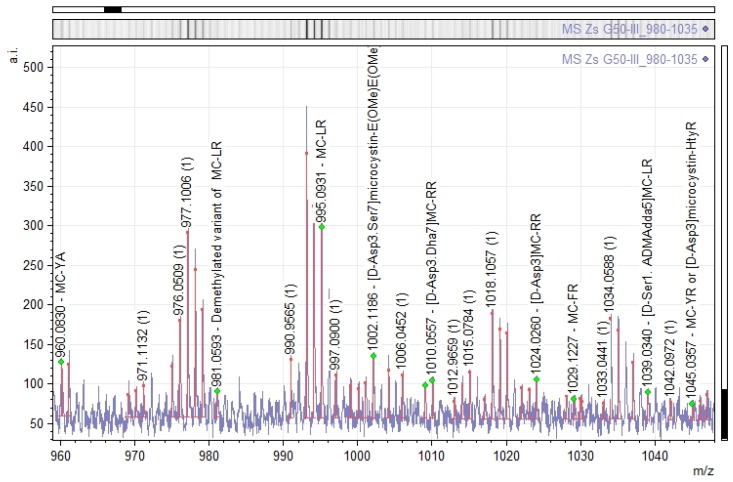
The figure shows absolute intensity (a.i) versus mass-to-charge ratio (*m*/*z*) in the *m*/*z* range 960–1050 from the MS analysis of fraction Zs G50-III. Some masses are highlighted as annotations, considering similar *m*/*z* matches as possible microcystins.

Moreover, some microcystin-related *m*/*z* signals were detected in the MALDI-TOF analysis of Zs G50-III within the *m*/*z* range 960–1050 (Figure 4). These signal are *m*/*z* 960.0830, microcystin YA (MC-YA) [33]; *m*/*z* 981.0593, demethylated variant of MC-LR ([DMAdda5]microcystin-LR) [32]; *m*/*z* 995.0931, MC-LR (microcystin-LR) [34]; *m*/*z* 1002.1186, [D-Asp3.Ser7]microcystin-E(OMe)E(OMe) [35] or microcystin LY (MC-LY) [36]; *m*/*z* 1010.0557, [D-Asp3.Dha7]microcystin-RR [37]; *m*/*z* 1024.0260, [D-Asp3]microcystin-RR [38]; *m*/*z* 1029.1227, microcystin FR (MC-FR) [39]; *m*/*z* 1039.0340, [D-Ser1. ADMAdda5]microcystin-LR [40]; *m*/*z* 1045.0357, microcystin YR (MC-YR) [34] or [Dha7]microcystin-HtyR [41]. However, these *m*/*z* signals were not successfully sequenced by MS/MS and, subsequently, analytical steps were conducted to corroborate the presence of microcystins.

### 2.3. Zs G50-III Reserved-Phase Chromatographic Analysis

#### 2.3.1. RP-HPLC Analytical Profile

**Figure 5:**
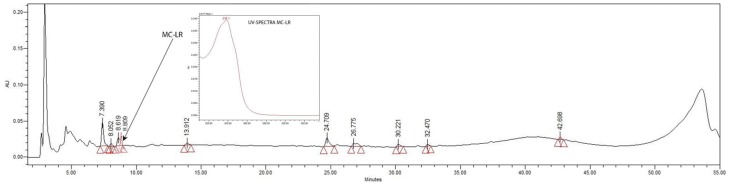
Analytical profile of fraction Zs G50-III, obtained by RP-HPLC. Gradient elution started from 1% to 99% MeOH over 55 min. The injected volume was 10 µL at a concentration of 1 mg/mL. The PDA range was 210–400 nm, with fixed wavelengths at 220 nm and 280 nm. The peak of the commercial standard of MC-LR (in red line) eluted at a retention time of 8.8 min. The injected volume was 10 µL at a concentration of 1 mg/mL. The UV spectrum of MC-LR shows maximum absorbance at 238 nm, typical of microcystins.

The detection of some microcystin-related *m*/*z* signals, prompted the performance of an analytical chromatographic step to ensure the identification of microcystins. Taking into account that the signal *m*/*z* 995.09 matched MC-LR, as the most commonly known and widely distributed microcystins, efforts were focused on confirming the presence of MC-LR in the sample. Therefore, the fraction Zs G50-III was first analysed by RP-HPLC in analytical mode to obtain preliminary information on the sample complexity. The analytical chromatogram showed what we considered as nine peaks of moderate intensity, where the highest relative absorbance was produced by the less retained compounds (Figure 5). Additionally, some of the peaks detected in the analytical chromatogram of the fraction Zs G50-III produced UV spectra with a λmax at 220 and 274 nm, while the other peaks’ UV maximum-absorbance were around 233, 260, and 300 nm (Supplementary Figure S1), close to the maximum absorbance of microcystins.

In addition, a chromatogram was obtained with a commercial standard of MC-LR in analytical mode in the same conditions as mentioned for fraction Zs G50-III (Figure 5). The elution time for the MC-LR standard was 8.8 min, whereas in the analytical mode three closely-eluting peaks at 7.3, 8.0, and 8.6 min were detected. Unlike MC-LR, such peaks do not show maximum absorbance at 238 nm (Supplementary Figure S1). This behaviour could be explained by insufficient separation of the components. 

#### 2.3.2. Zs G50-III RP-HPLC Semi-Preparative Assays

**Figure 6:**
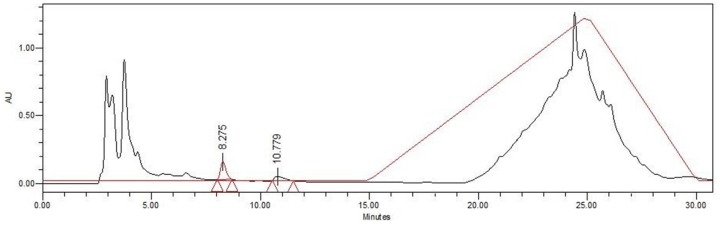
Chromatogram of fraction Zs G50-III subjected to reversed-phase C18 HPLC in isocratic mode with 10% MeOH using a 0.1% TFA/H_2_O/MeOH elution system. After 15 min the solvent was increased from 10% to 99% MeOH over 30 min. The chromatogram obtained by the RP-HPLC procedure shows relative absorbance at 220–280 nm of two peaks and their respective retention times of 8.2 min for peak 1 and 10.7 min for peak 2.

Afterwards, a semi-preparative RP-HPLC run (Figure 6) was carried out to separate those components with retention time like that of the commercial MC-LR chromatogram (Figure 5). Unlike microcystins, the UV maximum absorbance for the first peak was 264 nm, whereas the second peak showed a UV maximum absorbance at 220–274 nm (Supplementary Figure S2), resembling a typical UV spectra of peptides [42,43,44,45]. In contrast to Liquid chromatography combining multi-stage mass spectrometry (MC-LC/MS), UV-based methods do not provide unequivocal identification of known, unexpected and/or trace levels of microcystins [46]. 

Nonetheless, the UV spectra of the peaks were not clearly related to MC-LR. Other cyclopeptides with similar elution time, maximum UV absorbance at 220–274 nm and *m*/*z* 995 were previously reported as cyanopeptolin [47]. However, the isomerization [48] and co-elution with another congeners, like cyanopeptolin [47,49] and microginin [50], can produce unusual UV spectra. The co-occurrence of cyanotoxins with other congeners is a common fact supporting the possibility of components co-elution. It is noteworthy the presence of putative co-eluting components detected by MS of the peak 2 (Supplementary Figure S3). Unfortunately, the amount of the fraction was limited, and further purification steps based on the gradient elution with phosphoric acid as the mobile phase was not possible to perform as previously reported [50].

### 2.4. Identification of MC-LR by MALDI-TOF/TOF Analysis

**Figure 7:**
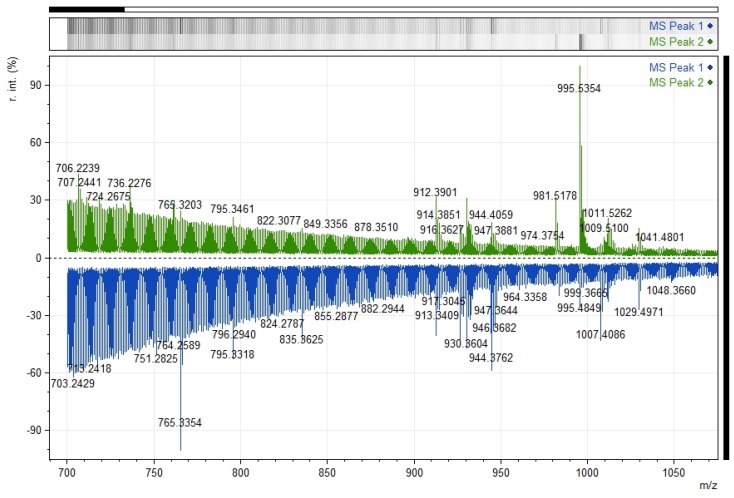
Matrix-assisted laser desorption/ionization time-of-flight/time-of-flight (MALDI-TOF/TOF) mass spectrum of the two RP-HPLC peaks. The composite figure shows relative intensity (r.int) versus mass-to-charge ratio (*m*/*z*) in the range of *m*/*z* 700–1075 of peak 1 (blue) peak 2 (green).

**Figure 8:**
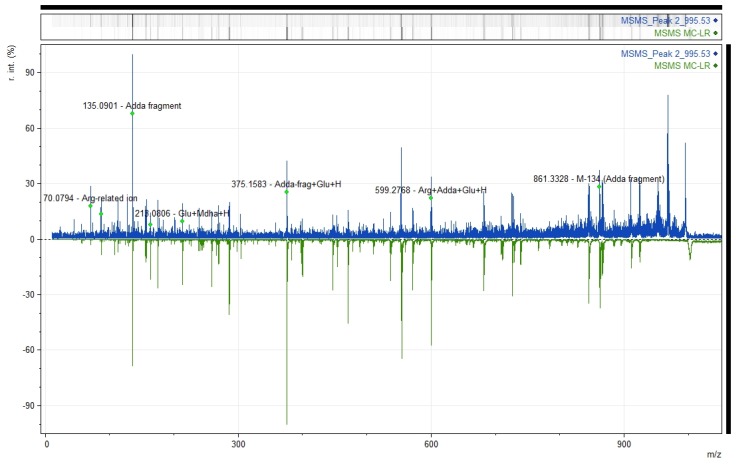
The figure shows relative intensity (r.int) versus mass-to-charge ratio (*m*/*z*) or the spectrum generated by MALDI-TOF/TOF of the signal *m*/*z* 995.53 from peak 2 (color blue, top panel) and the MS/MS of 1 µg of an MC-LR commercial standard (blue, bottom panel). Note similarities in the fragmentation pattern in both spectra; the most relevant ions in the identification of the MC-LR are annotated in the top panel.

The two peaks obtained by RP-HPLC were first submitted to MALDI-TOF MS scan resulting in the detection of *m*/*z* values common to both peaks, such as *m*/*z* 795.3, 912.3, 926.4, 930.3; 944.4; 995.5, 1007.4, 1011.5, and 1029.5 (Figure 7). Nonetheless, some differences were detected in the peak list and signal intensity, among which the *m*/*z* 765.33 showed the highest intensity in peak 1, whereas 995.53 was the most intense *m*/*z* signal in peak 2. The mentioned *m*/*z* 995.53 was successfully sequenced via MS/MS analysis of peak 2 (Figure 8) and the resulting spectrum matched the *m*/*z* values of some fragments produced by MS/MS analysis of MC-LR (Figure 8). Some fragments from the MS/MS of *m*/*z* 995.53 were successfully annotated, including *m*/*z* 135, as a diagnostic characteristic of microcystins. Some of the fragments detected were annotated, such as *m*/*z* 70.07 [Arg-related ion], *m*/*z* 135.09 [Adda fragment], *m*/*z* 375.15 [Adda-fragment + Glu + H], 599.27 [Arg + Adda + Glu + H], and 861.33 [M-134]. Others *m*/*z* signals, like 1224.59, 1471.68, 1731.82, and 1866.87, corresponding to co-eluting components to the MC-LR (*m*/*z* 995.53), were also successfully fragmented on peak 2, (Supplementary Figure S3). Although, it was not possible to identify them, the *m*/*z* signal 1731.82 and 1866.87 resembled microviridins previously described [51].

### 2.5. Putative Origins of Microcystins in the Zs G50-III Fraction

Cyanobacteria also known as blue-green algae are a widely distributed group of photosynthetic prokaryotic organisms [32,52,53]. Some genera like *Microcystis*, *Planktothrix*, *Anabaena*, *Nostoc*, and *Nodularia* can produce diverse toxins called cyanotoxins [32]. Within them, the microcystins mostly produced by *Microcystis aeruginosa*, comprise more than 100 variants of such cyanotoxin [54]. The microcystins are synthesized via a non-ribosomal pathway, where peptide synthetases (NRPS) and polyketide synthases (PKS) play an important role [55]. The general structural of these cyclic heptapeptides are: cyclo [D-Ala(1)–L-X(2)–DMeAsp(3)–L-Z(4)–Adda(5)–D-Glu(6)–Mdha(7)], where L-X and L-Z in position 2 and 4 of the ring are variable L-amino acids, D-MeAsp is a non-proteogenic aminoacid D-erythro-b-methylaspartic acid, Mdha is N-methyldehydroalanine [56]. The Adda, that is (2S,3S,8S,9S)-3-amino-9-methoxy-2,6,8-trimethyl-10-phenyldeca-4,6-dienoic acid, is a diagnostic characteristic of microcystins. Each microcystin is named depending on the identity of X and Z amino acids [30].

Cyanobacteria toxins are more common in world-wide freshwater ecosystems [57,58], although cyanobacteria are widespread in estuarine and marine systems, as well [59,60]. However, there are reports on the occurrence of microcystins in marine ecosystems producing hepatic necrosis, haemorrhage, and sudden death in marine mammals [23,61]. The most significant report produced by microcystins intoxication produced the death of 21 southern sea otters [23]. The land-sea flow with trophic transfer through the marine invertebrate-producing bioaccumulation process was demonstrated. It is noteworthy that our specimens belong to the invertebrate *Z. sociatus*, sampled at the Quibú river mouth, in Havana City. Moreover, this river passes through the city and is likely to be eutrophyzed, providing the proper conditions for the occurrence of microcystins.

Additionally, the confirmation of microcystin-contaminated freshwater outflows to the ocean is not an isolated and sporadic event [23,24,25,26,27]. There is also evidence of microcystins in some marine animals, like copepods, corals, and fish [28,62,63]. In addition, bioaccumulation of microcystins have been detected on fresh and saltwater mussels [64,65], farmed crustaceans [66,67], fishes [63], and probably in humans [68]. In our case, the cyanobacteria-producing toxins should come from the river flow to the marine zone, occurring as a guest of *Z. sociatus* until being fed. Then microcystins can be incorporated into the *Z. sociatus* tissues. Therefore, we suggest that the most probable source of the toxins detected in our sample is an external producer and not a product of the zoanthid *Z. sociatus*. However, the hypothesis of *Z. sociatus* as responsible for the production of microcyistin should not be completely discharged, since an analogue of microcystins, called motuporin (analogue of Nodularin-R), was first isolated and described in the marine sponge *Theonella swinhoei* [69].

Taking into account the high toxicity of microcystins on mammals, specifically in mice [70], the presence of microcystins in the studied fraction could be related to the lethal effects on mice previously reported of a LMW fraction from *Z. sociatus* [19]. In fact, MC-LR and its congeners are highly toxic in mice (lethal dose: LD_50_ = 50 µg/kg) when administered intraperitoneally [71]. Unlike Zs G50-III, mostly of the known MC-LR toxic effects are hepatotoxic. In addition, the mentioned studies lack of enough elements to know if MC-LR was tested in an acute toxicity assay producing death in a short time. Nonetheless, the possibilities of MC-LR be involved in cardiotoxic effects could not be discharged, since it is capable of modulating Ca^2+^ channels. Indeed, the presence of microcystins on the fraction Zs G50-III could also be related to the effects on insulin secretion mediated by Ca^2+^ influx blockade in isolated rat β cells [18]. In fact, exposure to MC-LR for 72 h suppresses cell viability, disturbs glucose-stimulated insulin secretion, and decreases the expression of insulin protein [72]. Although it is not clear if the presence of microcystins are responsible for the mentioned effects, they are probably largely involved.

## 3. Conclusions

The present work represents one of the few attempts to identify new peptide toxins from the zoanthid *Z. sociatus*. However, the mass spectrometry analysis of a fraction from *Z. sociatus* resulted in the detection of masses below 2000 Da, among which none sea anemones-like toxins were detected with the Protein Pilot protein identification software and Mascot algorithm search. Nonetheless, some related microcystins masses were detected within the most intense signals generated by the mass spectrometry analysis. One of them, *m*/*z* 995.53 resulted in a highly similar fragmentation pattern than that of MC-LR standard. Most of the fragment produced by the ionization of the *m*/*z* 995.53 were successfully annotated, including *m*/*z* 135.09 [Adda fragment], as a diagnostic characteristic of microcystins. The combination of the evidences detected led to confirm the presence of MC-LR in the fraction Zs G50-III. To date, this finding constitutes one of the few pieces of evidence of such cyanotoxin being detected in cnidarians, and the first report in the zoanthid *Z. sociatus*. In the cases of *m*/*z* 1066.00 and 1050.04, we considered that the information provided by the MS/MS spectra is insufficient to identify these signals as coming from microcystin [9-acetyl-Adda5]-MC-RR and MC-(H_4_) YR, respectively. Additionally, the presence of microcystins in fishing areas with similar conditions to that of the *Z. sociatus* sampling area remain unexplored in Cuba. Considering the mentioned evidence, the occurrence of microcystins in fishes should be of high probability around the sampling place. Therefore, these findings clearly suggest the underestimated risk of intoxication by microcystins occurring in water and food in a tropical region.

## 4. Materials and Methods

### 4.1. Preparation of Crude Extract and Gel Filtration on Sephadex G-50

The preparation of the *Z. sociatus* crude extract was performed from wild-caught colonies sampled at Quibú River mouth, in Havana, Cuba. The gel filtration chromatographic separation on Sephadex G-50 were performed as previously described [19]. Briefly, *Z. sociatus* specimens were blended after removing their stolonal bases. The whole-body homogenate was filtered through a spun glass mesh, and the filtrate was centrifuged twice in a Beckmann CS-6RK centrifuge at 1376× *g* for 30 min at 4 °C. The supernatant was freeze-dried overnight and then submitted to fractionation on a Sephadex G-50. The most prominent fraction of the chromatogram, called Zs G50-III was freeze-dried and stored for subsequent experiments.

### 4.2. Mass Spectrometry Analysis and Database Search

Mass spectrometry analysis was performed by MALDI-TOF/TOF (4800 Plus MALDI-TOF/TOF Analyzer; AB SCIEX, Framingham, MA, USA). Mass spectra were analysed with the Data Explorer software (v3.7, build 126, AB SCIEX, Framingham, MA, USA). The samples obtained in the semi-preparative HPLC-PDA separation, corresponding to peaks 2, 3, 4, 5, and 6, were co-crystallized at room temperature with the matrix α-cyano-4-hydroxycinnamic acid, α-CHCA at 10 mg/mL (50% acetonitrile, and 0.1% trifluoroacetic acid (TFA)) in a MALDI target plate. Samples were previously concentrated and cleaned according to the manufacturer’s instructions on a micro C18 column (ZipTip, Millipore, Bedford, MA, USA). The mass spectra were acquired in positive ion reflector mode from *m*/*z* 700 to 4000. Afterwards, some of these peptides peaks were selected for MS/MS peptide sequencing.

The masses [M + H]^+^ and the corresponding native intensity of HPLC-PDA peaks obtained from MS scan were exported to ASCII format for further analysis with the mMass 5.5.0 software [73,74]. Firstly, Peak Lists was achieved using the following criteria: peak’s signal-to-noise ratio threshold (S/N) 2.0; absolute intensity threshold (a.i) 10.0 (peak’s native intensity without baseline correction); relative intensity threshold (r.int.) 0.3 (peak’s relative intensity, in percentage of the most intense peak); and picking height of 75. Additionally, baseline correction, smoothing, and deisotoping tools were also applied to all MS fixed at default settings. The matrix α-CHCA monoisotopic masses [M + H]^+^ acquired in the same MS mass window were used as control for calibration. The resulting peak lists were compared to each other using a tolerance of 0.1 Da and different masses were determined. Finally, all different candidates’ masses [M + H]^+^ were corroborated in each MS (each MS-peak-spectrum against MS-matrix-spectrum) in both mode Autoscale and Normalize View. 

The data generated by MALDI-TOF/TOF procedure were submitted to search with the Mascot (Matrix-Science, London, UK) algorithm. The protein identification was carried out from UniProtKB protein sequence database [29] in the Metazoa and Cnidaria section. The Mascot search parameters were fixed as follows: up to two maximum trypsin missed cleavages, mass tolerance of 50 ppm, cysteine carbamidomethylation (fixed modification), methionine oxidation (variable modification), and a charge state of +1. The same data mentioned were also analysed by Protein Pilot protein identification software v4.5 (AB SCIEX). The protein identification was carried out from UniProtKB protein sequence database in the Metazoa and Cnidaria section. Only those proteins matched with scores at a 95% confidence level were considered as significant hits. 

Moreover, all spectra generated for each peak were also analyzed by a mass-matching tool provided by the mMass software v5.5.0 [73,74]. In this case, the analysis was carried out within the internal monomer mMass library and also against the imported non-ribosomal peptides library (NORINE peptide database, available in the mMass homepage at the download section http://www.mmass.org/download/), adapted from the original NORINE database [21]. The matched masses were then examined, and their spectra were analyzed. The fragmentation pattern was compared to a standard or available reference, in the case of those that were of interest. 

#### MALDI-TOF/TOF Raw Data Availability

The output files generated by MALDI-TOF/TOF analyses used in this work are provided in ASCII format as Supplementary Raw Dataset S1. Within the package are included the following folders containing the respective mass spectra (MS/MS): Matrix (MALDI TOF/TOF MS of matrix used as control; MS_Zs G50-III (MS of studied fraction Zs G50-III); MS/MS_1066_&_876 (MS/MS of the signals *m*/*z* 1066.00 and 876.94 from the MS of fraction Zs G50-III); MS/MS_1050_&_861 (MS/MS of the signals *m*/*z* 1050.04 and 861.01 from the MS of fraction Zs G50-III); Peak 1 (MS of Peak 1 was obtained by RP-PHLC from Zs G50-III fraction); Peak 2 (MS of Peak 2 was obtained by RP-PHLC from the Zs G50-III fraction, and the MS/MS of the signals *m*/*z* 995.53, 1224.59, 1471.68, 1731.82, and 18,866.87), and MS_&_MS/MS_MC-LR (MS of the commercial standard of MC-LR, and MS/MS of the signals *m*/*z* 995.53 and 981.50 were obtained from the MS of MC-LR).

### 4.3. Reserved-Phase Chromatographic Analysis

All solvents used in RP-HPLC analyses were of high-purity chromatography grade (LiChrosolv, Merck, Darmstadt, Germany). Aqueous solutions were prepared with ultrapure water supplied from a Millipore water purification system (0.0054 µS·cm^−1^). Trifluoroacetic acid (TFA) was of spectrophotometric grade 99%. The chromatographic system used for RP-HPLC separations was a Waters Alliance e2695 HPLC coupled with a photodiode array (PDA) 2998 and an automatic fraction collector. Empower Two Chromatography Data Software was used for calculation and reporting peak information. All HPLC solvents were filtered (Pall GH Polypro 47 mm, 0.2 μm) and degassed by an ultrasonic bath.

The analytical profile was obtained on a Merck Lichrospher RP-18 endcapped reversed-phase column (250 mm × 4.6 mm i.d., 5 µm) equipped with a guard column (4 × 4 mm, 5 µm), both kept at 40 °C. The PDA wavelength range was 210-800 nm with fixed values at 220 and 280 nm. The solvent system consisted of MilliQ water (H_2_O) and methanol (MeOH), both acidified with 0.1% TFA. A linear gradient from 1% to 99% MeOH was applied in the analytical run to explore the complexity of the sample and the elution time of the commercial standard of Microcystin-LR (DHI Water and Environment, Denmark; Batch No: MCLR-111).

The purification step was performed using a Phenomenex Luna RP-18 (25 cm × 10 mm, 10 μm) chromatographic column kept at 35 °C. The PDA range was 210–800 nm with fixed wavelengths of 220 and 280 nm. For the isocratic elution, 0.1% TFA in 10% MeOH was used as eluent at a flow rate of 2.5 mL/min. Eventually, the solvent of the mobile phase was increased to 50% MeOH. The concentration of total-proteins estimated was 1 mg/mL, and injected in a final volume of 200 µL. The fractions were automatically collected following the PDA signal. The fractions of interest were dried up by speed vac and kept at −20 °C for subsequent analysis.

## Supplementary Materials

The following are available online at www.mdpi.com/2072-6651/9/3/89/s1, Figure S1: The figure shows the UV spectra of peaks obtained from the RP-HPLC analytical profile of fraction ZsG50-III, Figure S2: The figure shows the UV spectra of two peaks obtained from the RP-HPLC of the fraction ZsG50-III in a semi-preparative mode, Figure S3: Matrix assisted laser desorption/ionization time-of-flight/time-of-flight(MALDI-TOF/TOF) mass spectra (MS) of the RP-HPLC Peak 2 and Supplementary Raw Dataset S1.

## Abbreviations

The following abbreviations are used in this manuscript:

LMW	Low molecular weight
MALDI-TOF/TOF	Matrix assisted laser desorption/ionization time-of-fly/time-of-fly
MS	Mass spectra
*m*/*z*	Mass-to-charge ratio
a.i	Absolute intensity
int	Peak’s corrected intensity
r.int	Relative intensity
S/N	Peak’s signal-to-noise ratio
mass	Neutral mass
fwhm	Full width at half maximum
resol	resolution
NORINE	Non-ribosomal peptide database
HPLC	High Performance Liquid Chromatographic
RP	Reverse Phase
MC-LR	Microcystin LR
*Z. sociatus*	*Zoanthus sociatus*
TTX	tetrodotoxin
*Palythoa caribaeorum*	*P. caribaeourum*
LC/ESI-Q-ToF	Liquid Chromatography/Electrospray ionization coupled to Quadruple Time-of-flight
Adda	3-amino-9-methoxy-2,6,8-trimethyl-10-phenyl-deca-4,6-dienoic acid
LD	Lethal Dose
UV	Ultraviolet
TFA	Trifluoroacetic acid
PDA	Photodiode Array
