# Supplementary material for: Microcystin-LR Detected in a Low Molecular Weight Fraction from a Crude Extract of Zoanthus sociatus
Source: Toxins (Basel). 2017 Mar 1;9(3):89. doi: 10.3390/toxins9030089 (PMC5371844; doi:10.3390/toxins9030089)
Supplement: Supplementary file 1 [file toxins-09-00089-s001.zip › toxins-159662 supplementary/toxins-159662 supplementary.pdf]

# Supplementary Materials: Microcystin-LR Detected in a Low Molecular Weight Fraction from a Crude Extract of *Zoanthus sociatus*

**Dany Domínguez-Pérez, Armando Alexei Rodríguez, Hugo Osorio, Joana Azevedo, Olga Castañeda, Vítor Vasconcelos and Agostinho Antunes**

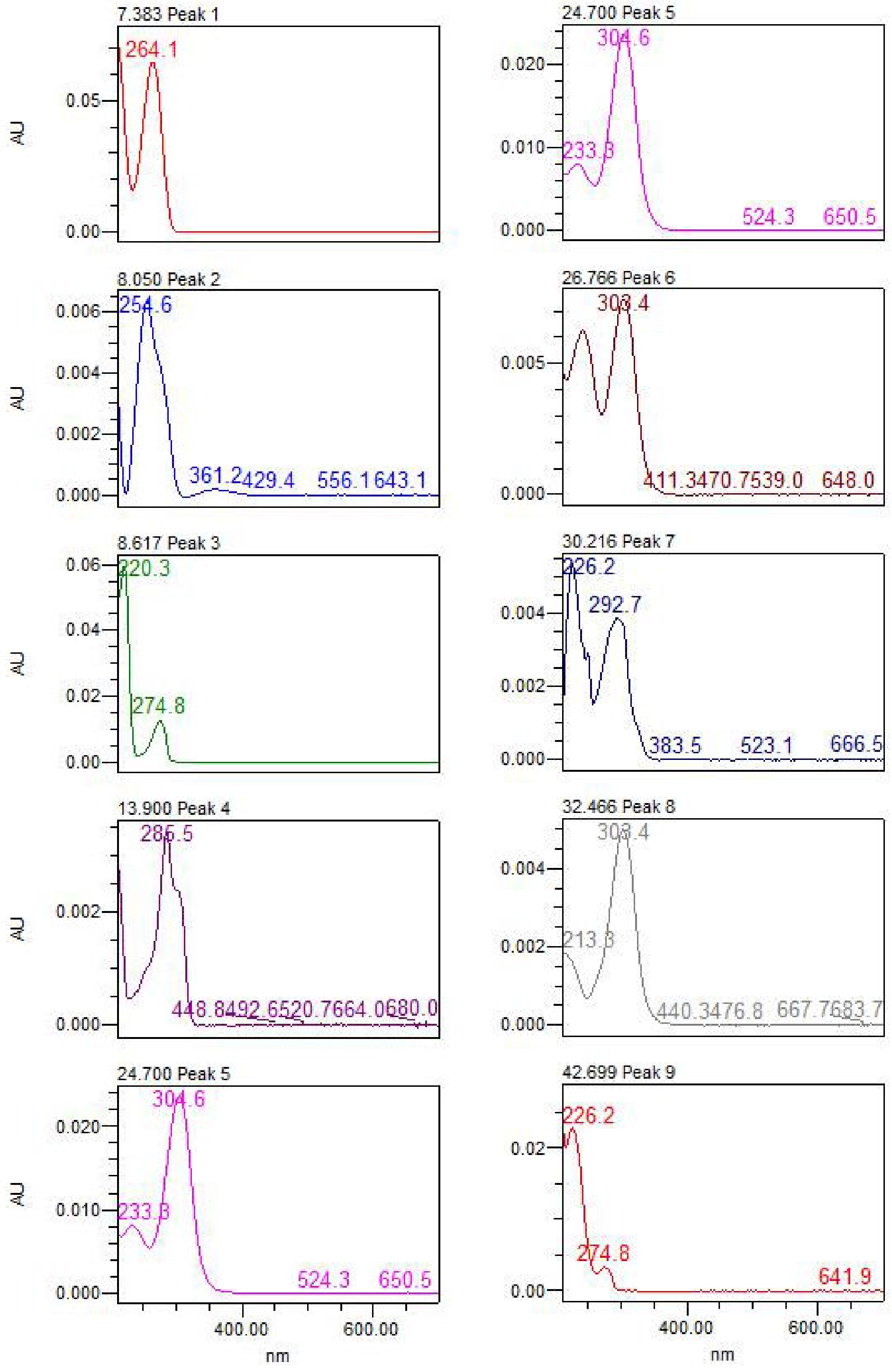


**Figure S1.** The figure shows the UV spectra of peaks obtained from the RP-HPLC aanalytical profile of fraction ZsG50-III. The maximum UV absorbance is variable within all peaks, within should be appreciated some peptides with UV spectra at 220–280, while another showed UV maximum at 233–304 nm.

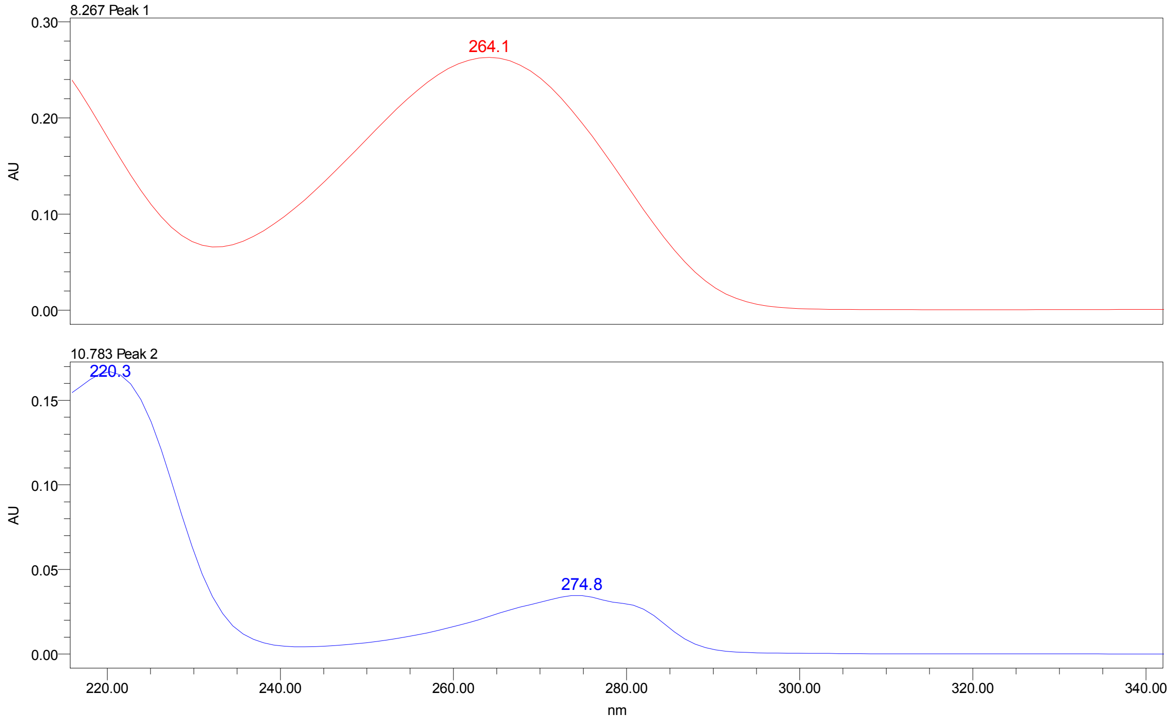


**Figure S2.** The figure shows the UV spectra of two peaks obtained from the RP-HPLC of the fraction ZsG50-III in a semi-preparative mode. The UV spectra showed maximum absorbance at 264 nm for peak 1, while UV maximum absorbance was 220–280 nm for peak 2.

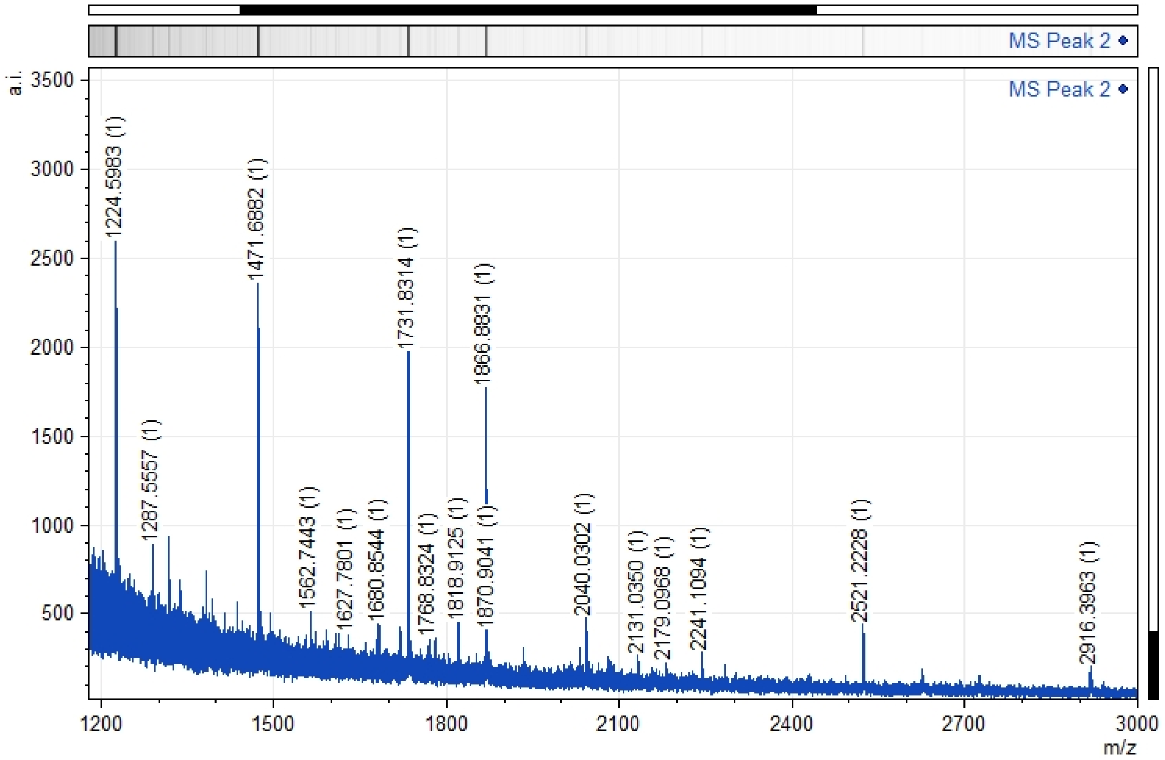


**Figure S3.** Matrix assisted laser desorption/ionization time-of-fly/time-of-fly (MALDI-TOF/TOF) mass spectra (MS) of the RP-HPLC peak 2. The MS spectra show absolute ion intensity (a.i) versus monoisotopic *m*/*z* value (*m*/*z*) of each peak 2 in a mass range from *m*/*z* 1180 to 3000.
